# Propolis Reduces the Expression of Autophagy-Related Proteins in Chondrocytes under Interleukin-1β Stimulus

**DOI:** 10.3390/ijms20153768

**Published:** 2019-08-01

**Authors:** Consuelo Arias, Nicolás Saavedra, Kathleen Saavedra, Marysol Alvear, Alejandro Cuevas, Silvya Stuchi Maria-Engler, Dulcineia S. P. Abdalla, Luis A. Salazar

**Affiliations:** 1 Center of Molecular Biology and Pharmacogenetics, Scientific and Technological Bioresource Nucleus, Universidad de La Frontera, Av. Francisco Salazar 01145, Temuco 4811230, Chile; 2 Preclinical Sciences Department, Faculty of Medicine, Universidad de La Frontera, Claro Solar 115, Temuco 4781218, Chile; 3 Department of Chemical Sciences and Natural Resources, Faculty of Engineering and Sciences, Universidad de La Frontera, Av. Francisco Salazar 01145, Temuco 4811230, Chile; 4 Department of Clinical and Toxicological Analyses, Faculty of Pharmaceutical Sciences, Universidade de São Paulo, Av. Prof. Lineu Prestes 580, Cidade Universitária-Butantã, São Paulo 05508-000, Brazil

**Keywords:** propolis, autophagy, chondrocytes

## Abstract

Background: Osteoarthritis (OA) is a progressive and multifactorial disease that is associated with aging. A number of changes occur in aged cartilage, such as increased oxidative stress, decreased markers of healthy cartilage, and alterations in the autophagy pathway. Propolis extracts contain a mixture of polyphenols and it has been proved that they have high antioxidant capacity and could regulate the autophagic pathway. Our objective was to evaluate the effect of ethanolic extract of propolis (EEP) on chondrocytes that were stimulated with IL-1β. Methods: Rabbit chondrocytes were isolated and stimulated with IL-1β and treated with EEP. We evaluated cell viability, nitric oxide production, healthy cartilage, and OA markers, and the expression of three proteins associated with the autophagy pathway LC3, ATG5, and AKT1. Results: The EEP treatment reduces the expression of LC3, ATG5, and AKT1, reduces the production of nitric oxide, increases the expression of healthy markers, and reduces OA markers. Conclusions: These results suggest that treatment with EEP in chondrocytes that were stimulated with IL-1β has beneficial effects, such as a decrease in the expression of proteins associated with autophagy, MMP13, and production of nitric oxide, and also increased collagen II.

## 1. Introduction

Osteoarthritis (OA) is a progressive, degenerative, and multifactorial joint that is disease characterized by a progressive loss of articular cartilage, subchondral bone sclerosis, osteophyte formation and synovial inflammation that has been positioned as the world-wide leading cause of pain and dysfunction [1,2,3,4,5,6,7]. Moreover, the high prevalence of OA and its great impact on the work ability make this disease an important social problem [8,9].

Aging is one of the most important risk factors of OA [5,6,10,11,12]. In aged cartilage as well as in OA, there is an increased production of reactive nitrogen and oxygen species (RNOS) that impact the adult chondrocytes, since they become more susceptible to cell death mediated by RNOS [13,14]. Chondrocytes are stimulated by proinflammatory cytokines, such as IL-1β, which leads to the production of large amounts of nitric oxide (NO) through the increased activity of inducible nitric oxide synthase (iNOS) [15,16,17]. Subsequently, NO inhibits the production of an extracellular matrix (ECM) and interferes with important paracrine and autocrine factors that are involved in OA, perpetuating the catabolic state of articular chondrocytes [15,16]. Animal and human articular cartilage with OA have both been reported to increased RNOS production, which also contributes to the degradation of cartilage by upregulation of matrix metalloproteinases (MMPs) such as MMP13 [18,19,20,21]. Moreover, the cellular oxidation caused by an increased production of RNOS leads to cell aging, especially in postmitotic tissues, such as cartilage [22], which depend on autophagy as the main mechanism for eliminating damaged or dysfunctional organelles and macromolecules [23].

Autophagy is a preserved evolutionary pathway of intracellular degradation, in which damaged organelles and long-lived proteins are degraded and recycled to maintain cellular homeostasis [24,25,26]. This process consists of dynamic membrane rearrangements that are mediated by a group of four main autophagy-related proteins (ATG) that include unc-51, like autophagy activating kinase 1 (ULK1), Beclin1 (BECN1), microtubule associated protein 1 light chain 3 alpha (LC3), and autophagy related 5 (ATG5). Upstream, the PI3/AKT and ERK/MAPK pathways are able to regulate the mammalian target of rapamycin (mTOR), which is a vital regulator of autophagy, by controlling the interaction between mTOR serine and threonine kinases in the mTOR complex 1 (mTORC-1) [27]. The inhibition of mTORC-1 promotes autophagy, while the activation of mTOR kinase suppresses it [27,28]. In addition to its key role in physiological conditions, aging is often accompanied by defects of general autophagy [29] and its deregulation is implicated in various pathological conditions, such as aging-related diseases [30,31]. In fact, alterations of autophagy are correlated with cell death and OA [31].

Autophagy has a controversial role in cellular survival and death [32,33]. Although autophagy mostly protects cells allow their adaptation to several types of stress, excessive or prolonged activation of this pathway can promote cell death [26,34]. Moreover, the autophagy pathway may be related to proinflammatory signaling through a mechanism that involves oxidative stress [35,36,37,38]. It has been observed that cellular damage that is generated by excessive production of RNOS can stimulate this pathway [22,26,39]. For this reason, the functional relationship between autophagy and apoptosis is complex and apparently it is the stimulus that determines an induction of apoptosis or autophagy in a context-dependent mode [34,40,41]. One of the links between these processes could be ATG5, which has a dual role in autophagy and apoptosis, because it could trigger apoptosis through several mechanisms and it is part of the molecular mechanisms that govern the inhibitory crosstalk between apoptosis and autophagy [40].

Although several therapeutic strategies have been developed to improve the repair of hyaline cartilage, none has been sufficiently effective to generate functional and long-lasting tissue. Currently, there are no drugs available to modify OA and a large number of candidate drugs have failed to demonstrate efficacy or they were associated with significant side effects [23,42]. This makes it necessary to search for other therapeutic alternatives that can avoid undesired effects and can be adapted to the progressive and multimodal nature of OA.

Polyphenols are the most common bioactive natural products that are present in fruits, vegetables, seeds, among others [43,44,45,46], and they have a wide range of activities in the prevention and treatment of various physiological or pathophysiological states, such as cancer, neuroinflammation, diabetes, and aging [47,48,49]. Several of the beneficial effects of polyphenols have been attributed to their antioxidant capacity and their ability to modulate antioxidant defense mechanisms [50]. Additionally, these bioactive components have a great potential to prevent diseases through genetic and epigenetic modifications [48,49,51,52,53]. Paullauf et al., also reported that polyphenols affect numerous cellular targets that can induce or inhibit autophagy and mention that autophagy interferes with the symptoms and putative causes of aging [54,55]. In fact, several studies described the regulation that polyphenols have on the path of autophagy [49,56,57,58,59,60].

Propolis extract is an extremely complex mixture of natural substances; it contains amino acids, phenolic acids, phenolic acid esters, flavonoids, cinnamic acid, terpenes, and caffeic acid [61], and it has multiple pharmacological properties, including hepatoprotective, antioxidant, and anti-inflammatory actions, and in the cartilage, it has been shown to offer excellent protection, being mediated in part by its RNOS scavenger action [62,63]. Pinocembrin (PB) is one of the most abundant flavonoids in propolis [64,65,66] and it has been associated to the inhibition of MMP-1, MMP-3, and MMP-13 expression at both the protein and mRNA levels in cartilage [66]. Additionally, it is suggested that PB could protect the brain against ischemia-reperfusion injury, and the possible mechanisms might be attributed to the inhibition of apoptosis and reversed autophagy disfunction in the penumbra area [65,67].

Altogether, this evidence suggests a potential effect of propolis in reverting the alterations on the autophagy pathway in chondrocytes with OA, promoting the viability of the chondrocytes and the maintenance of healthy cartilage. Thus, the goal of our study was to evaluate the effect of ethanolic extract of propolis (EEP) in chondrocytes that were stimulated with IL-1β and its influence on the expression of proteins related to autophagy pathway and healthy and osteoarthritic marker.

## 2. Results

### 2.1. Characterization of Polyphenols Present in the EEP

**Figure 1:**
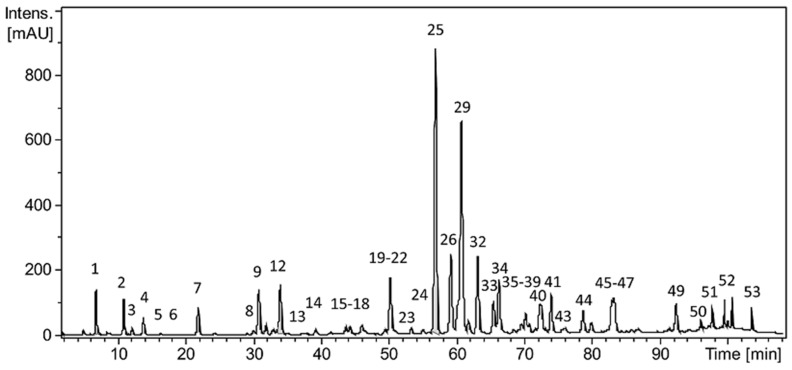
Characterization of polyphenols present in the Ethanolic Extract of Propolis. Chromatogram at 290 nm showing 53 peaks of compounds detected in ethanolic extract of propolis (EEP).

The chromatographic profile of the EEP was obtained while using HPLC, showing about 53 peaks (Figure 1), whose identification was assigned by analyzing standards using the same conditions as in EEP sample, while considering the exact mass, UV absorption spectrum, and decomposition in the gas phase (fragmentation). Table 1 describes the identified compounds and related data. Additionally, the amount of PB was measured by mass spectrometer, resulting in a concentration of 44.1 mg mL^−1^.

### 2.2. Cell Viability Analysis after Treatments

**Figure 2:**
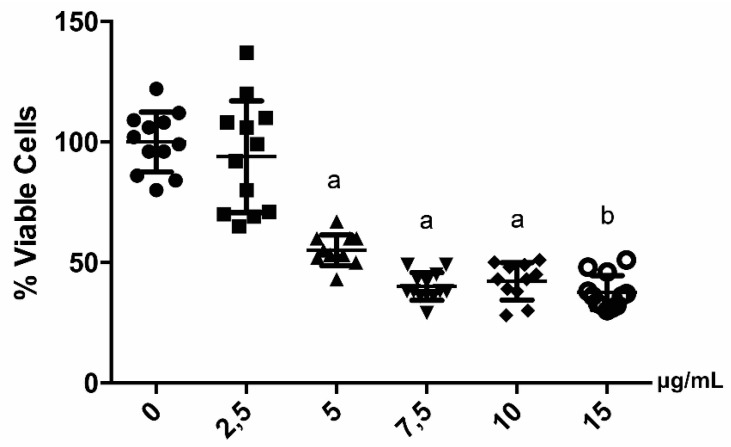
Cell viability analysis of Chondrocytes after EEP treatment. Quantification of cell viability with trypan blue, after application in the supernatant of different doses of propolis for 24 h in culture of rabbit chondrocytes in P2. (*N* = 12 technical replicates). Different letters show statistically significant differences compared to the untreated group (0μg/mL), a: *p* ≤ 0.05 and b: *p* ≤ 0.01.

Cell viability assay by trypan blue was performed to identify the highest dose of EEP that does not show a significant decrease in chondrocytes viability. After the EEP treatment using concentrations that ranged between 0 and 15 µg/mL, a significant decrease in cell viability was observed, starting from 5 µg/mL (Figure 2). For this reason, 2.5 µg/mL was selected as the dose for the following EEP treatments. The IL-1β, bafilomycin, and rapamycin doses were selected according to the literature and these did not significantly modify the viability of the chondrocytes (data not shown).

### 2.3. Effect of OA Induction on Autophagy-Related Proteins

**Figure 3:**
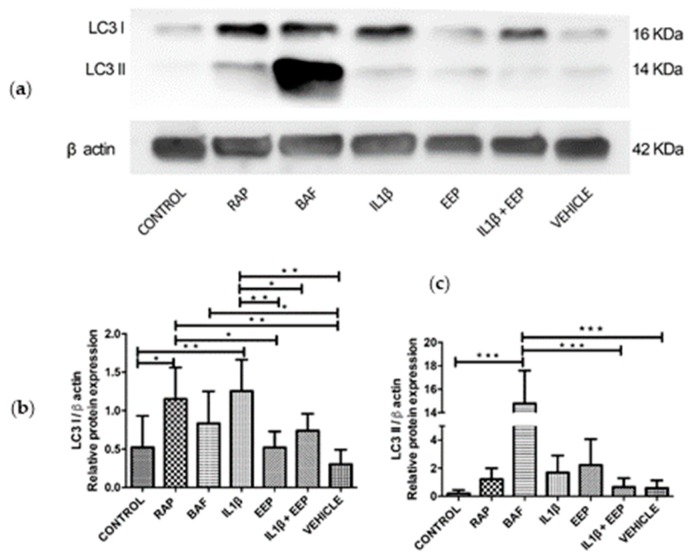
Effects of IL-1β and EEP treatment on autophagy markers. (**a**) Relative expression of LC3 I and II proteins evaluated by Western Blotting. (**b**,**c**) Quantification of protein expression of LC3 I and II evaluated in rabbit chondrocytes exposed to different condition per 24 h. Control (No treatment); RAP (100 nM rapamycin); BAF (20 nM bafilomycin); IL-1β (10 ng/mL IL-1β); EEP (EEP 2.5 μg/mL); IL-1β and EEP (10 ng/mL IL-1β and 2.5 μg/mL EEP) and vehicle (2% ethanol). * *p* < 0.05, ** *p* < 0.005, *** *p* < 0.001. (*N* = 5 independent experiment).

LC3I and LC3II proteins were detected by western blot (Figure 3a) to evaluate the effect of IL-1β-induced OA on autophagy pathway. An increase in the protein expression of LC3I was observed under IL-1β inflammatory stimulus, similarly to that observed after the treatment with rapamycin (RAP), which corresponds to a well-known autophagy stimulator (Figure 3a,b). Subsequently, no changes in the protein expression of LC3I or LC3II were observed when EEP or vehicle treatment was applied (Figure 3b,c). The cells exposed to bafilomycin (BAF), an autophagy inhibitor, markedly increased LC3II expression (Figure 3a,c). This accumulation in response to BAF treatment was described in the guidelines for the use and interpretation of assays for the monitoring autophagy by Klionsky et al. [7], where the accumulation of LC3I and II can be obtained by interrupting the autophagosome-lysosome fusion step, increasing the number of autophagosomes. Finally, a decrease in LC3I was observed in OA-induced chondrocytes after EEP treatment when compared to cells stimulated with IL-1β (Figure 3a,b).

### 2.4. Effect of EEP Treatment on Autophagy Protein in OA Chondrocytes

**Figure 4:**
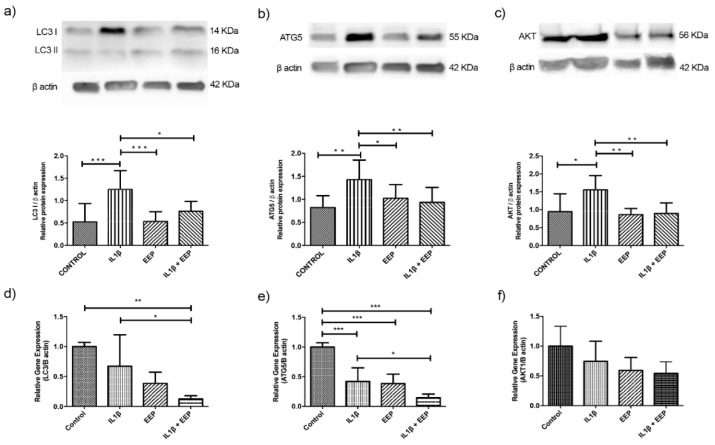
Relative expression of autophagy-related genes and proteins from rabbit chondrocytes stimulated by IL-1β and treated with EEP for 24 h. (**a**–**c**) Protein expression of LC3 I, ATG5, and AKT1 evaluated by Western Blotting and quantitative analysis. (**d**–**f**) Gene expression analysis of markers of the autophagy pathway evaluated by RT-PCR. * *p* < 0.05, ** *p* < 0.005, *** *p* < 0.001. IL-1β: stimulated with IL-1β 10 ng/mL, EEP: treated with EEP 2.5 μg/mL, IL-1β EEP: treated with IL-1β and EEP. All data were normalized by control (without treatment). One-way ANOVA, Tukey Multiple Comparison Test. SW normality test. (*N* = 5 independent experiment).

To analyze the autophagy pathway in OA chondrocytes under EEP treatment, three proteins were selected: LC3, ATG5, and AKT1. The protein expression of LC3I had a significant decrease in the condition co-treated with IL-1β and EEP when compared to the IL-1β group, and there were no significant differences between EEP treatment, and the EEP and IL-1β condition (Figure 4a). In relation to LC3 gene expression, a significant decrease was observed in OA chondrocytes that were treated with EEP, as compared to the IL-1β stimulated condition (Figure 4d).

Regarding ATG5 protein expression, a significant decrease was observed in the condition of co-treated with IL-1β and EEP when compared to the IL-1β group and there were no significant differences between EEP treatment, and EEP and IL-1β condition (Figure 4b). In relation to ATG5 gene expression, a significant decrease was observed in OA chondrocytes that were treated with EEP compared to IL-1β stimulated condition (Figure 4e).

Finally, a significant decrease of AKT1 protein expression was observed in the condition co-treated with IL-1β and EEP when compared with the IL-1β group and there were no significant differences between EEP treatment, and EEP and IL-1β condition (Figure 4c). This effect was not reflected in the gene expression analysis (Figure 4f).

### 2.5. Effect of EEP Treatment on Cartilage Markers Expression in OA Chondrocytes

**Figure 5:**
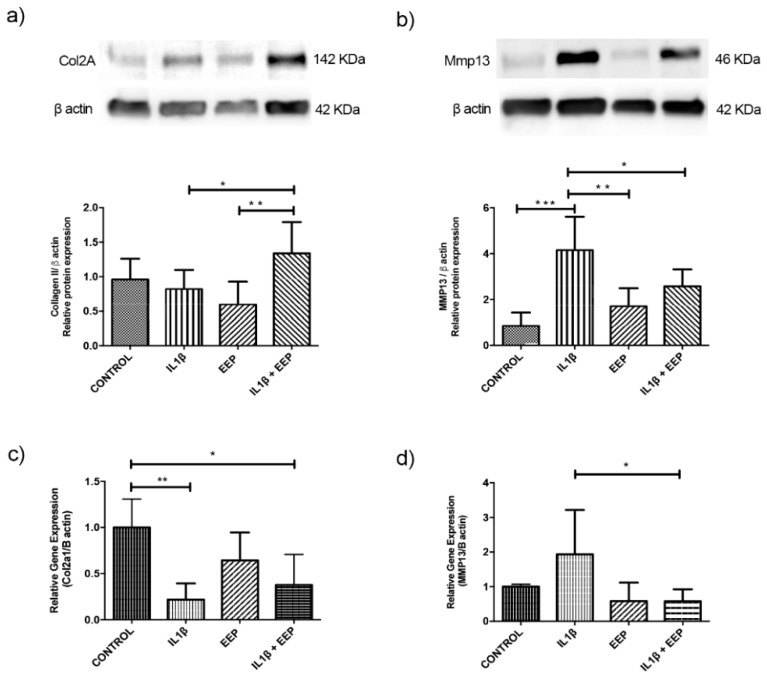
Relative protein and gene expression of cartilage markers in rabbit chondrocytes in response to IL-1β stimulation and EEP treatment for 24 h. (**a**,**b**) Protein expression evaluated by Western Blotting of col2a1 and MMP13 and quantitative analysis. (**c**,**d**) Relative expression of mRNA levels of col2a1 and MMP13 evaluated by RT-PCR. * *p* < 0.05, ** *p* < 0.005, *** *p* < 0.001. IL-1β: stimulated with IL-1β 10 ng/mL, EEP: treated with EEP 2.5 μg/mL, IL-1β EEP: treated with IL-1β and EEP. All the results were normalized by control (without treatment). One-way ANOVA, Dunnets and Tukey Multiple Comparisons (*N* = 5 independent experiments).

Collagen II (Col2a1) was selected as a healthy cartilage marker and MMP13 as OA marker. Col2a1 protein expression did not significantly change with EEP treatment when compared to the IL-1β stimulation. However, there is a significant increase from 1 to 1.4 in the condition of being co-treated with IL-1β and EEP when compared to the IL-1β stimulation, and between EEP, and IL-1β and EEP (Figure 5a). On the other hand, a significant increase in gene expression is observed between the IL-1β and EEP condition, but not in the condition of being co-treated with IL-1β and EEP (Figure 5c).

There is a significant decrease in MMP13 protein expression when EEP treatment is co-treated with IL-1β stimulation as compared to the IL-1β condition and there were no significant differences between EEP treatment, and EEP and IL-1β co-treated condition (Figure 5b). In relation to gene expression, there is a significant decrease in the EEP and IL-1β co-treatment when compared to the IL-1β condition (Figure 5d).

### 2.6. Effect of EEP Treatment on Chondrocytes Nitric Oxide Production Induced by the Inflammatory Stimulus

**Figure 6:**
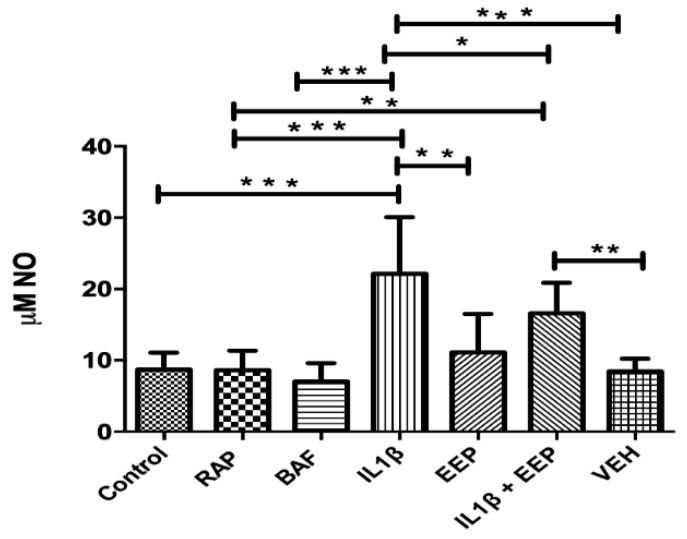
Analysis of nitric oxide in samples of rabbit chondrocytes stimulated with IL-1β and treated with EEP for 24 h. Different letters indicate statistic differences significance. Control: No treatment, Control RAP: treated with rapamycin 100 nM (positive control autophagy), Control BAF: treated with bafilomycin 20 nM (negative control autophagy), IL-1β: treated with IL-1β 10 ng/mL, EEP: treated with EEP 2.5 μg/mL, IL-1β EEP: treated with IL-1β and EEP and vehicle: treated with ethanol. * *p* < 0.05, ** *p* < 0.005, *** *p* < 0.001. One-way ANOVA, Tukey Multiple Comparison Test. (*N* = 5 independent experiments).

A significant increase in the amount of NO from 8 to 22 μM was observed in chondrocytes that were stimulated with IL-1β as compared to the control condition; this increase is reduced significantly to 16 μM with EEP treatment (Figure 6). In addition, EEP treatment does not modify the amount of NO in the supernatant compared to the control condition. On the other hand, the activation or inhibition of autophagy does not significantly modify the amount of NO that is released to the medium (Figure 6).

## 3. Discussion

OA is the most common chronic degenerative joint disease and it heavily impacts on life quality [8,9]. OA treatment is complex, because it is a multifactorial disease and current therapies are, at best, moderately effective pain relievers and several of these drugs have adverse effects [9], so the development of safe treatments is necessary.

Autophagy is a highly conserved mechanism of homeostasis maintenance [55,68] and its deregulation contributes to OA development. In fact, in late stages of OA, this process also could be conjunctly activated with apoptosis as an alternative pathway to chondroptosis [69,70]. Hence, the modulation of autophagy can be a promising therapeutic strategy for OA, since it has the potential to counteract both effects of the inflammatory stimuli and age-related defects [8].

Propolis is highly rich in active components and its extracts have numerous applications in treating various diseases [71]. PB is one of the most abundant flavonoid in propolis [65,72] and it has drawn much attention for its broad spectrum of pharmacological properties, such as the reversion of autophagy dysfunction in the ischemia-reperfusion injury [65,67]. We evaluated the amount of pinocembrin present in the Chilean propolis used in this study, since its composition depends on the source of the various trees that were used by honeybees [72,73]. The used EEP contains a significant amount of pinocembrin, being polyphenol the most abundant extract (Figure 1 and Table 1). These results are corroborated by previous studies of propolis that were extracted from the same region [74,75,76].

Given that autophagy defects could have a central role in OA, the objective of this study was to evaluate the potential effect of propolis in autophagy proteins on chondrocytes with OA. Importantly, there are no absolute criteria for determining autophagic status that are applicable in every biological or experimental context [7]. The radio between LC3I and LC3II is used to evaluate the autophagy pathway [55]. One limitation of our study was that it was not possible to calculate said radius due to the low abundance of LC3II, hence preventing reliable quantification. Therefore, it was decided to omit this indicator. Nevertheless, LC3I is used as a marker of autophagic induction [55] and the conversion pattern of LC3I to LC3II is dependent on the type and cellular context [7], so we evaluated the expression of this marker in response of IL-1β stimulation.

Certain studies have indicated that stimulation with IL-1β could inhibit the autophagy pathway, especially in those that are associated with nutrient deprivation [77]. In our study, we decided to observe what happens with the autophagy pathway without a context of nutrient deprivation.

After the IL-1β stimulation, there was an increase in LC3I protein expression similar to the one that was observed with the autophagy inducer, rapamycin (Figure 3). This suggests that the inflammatory stimulus with IL-1β increases the expression of LC3, which is consistent with the literature [78,79,80]. On the other hand, a decrease in LC3I protein expression with EEP treatment on OA chondrocyte was observed, as compared to chondrocytes that were stimulated with IL-1β, which suggested that EEP could regulate the autophagy pathway (Figure 3).

Subsequently, an analysis of gene and protein expression of three genes associated with the autophagy pathway was performed in response to IL-1β-stimulation and treatment with EEP in chondrocytes: LC3, ATG5, and AKT1. In response to IL-1β–stimulation, thee chondrocytes increased their protein expression of LC3I, ATG5, and AKT1 (Figure 4a–c). This result was also observed in mRNA expression of LC3I and ATG5 (Figure 4d,e), which suggested that the autophagy pathway is triggered by the inflammatory stimulus. Autophagy has been recognized as an adaptive response to stress that promotes survival, whereas in other cases it is capable of promoting cell death and morbidity [68,69,70], and a functional relationship between both processes is postulated [34,40,41]. This is also consistent with the effects of another proinflammatory cytokine, TNFα, which also increases with age and in OA, which is additionally able to increase the expression of LC3 through the inhibition of AKT activation [79,80]. AKT1 is an upstream regulator of mTOR and is usually considered a suppressor of autophagy, but it has been observed that some natural components activate autophagy with a concomitant increase in AKT phosphorylation [27]. 

On the other hand, it has been shown that the acute stimuli of oxidative stress may be able to induce a positive regulation of autophagy in an adaptive way, which helps to restore intracellular homeostasis, however, an alteration in autophagy may be generated if this stress is prolonged [35]. In aged cartilage and the development of OA, there is an increase ROS levels and adult chondrocytes become more susceptible to cell death that is mediated by ROS [13,14]. In addition, chondrocytes that are stimulated by proinflammatory cytokines, such as IL-1β, produce large amounts of nitric oxide [15,16,81,82], which contributes in part to this oxidative/nitrosative stress. This agrees with what we observed in chondrocytes that were stimulated with IL-1β (Figure 6). We suggest that the alterations produced in chondrocytes with OA are partly produced by this oxidative stress that is induced by NO.

Some polyphenols might regulate several cellular targets that can thus induce or inhibit autophagy [54], and it is thought that a partial restoration of basal autophagy might contribute to improving chondrocyte viability in OA [55]. Therefore, we evaluated whether the EEP, rich in polyphenols, could regulate the expression of the selected proteins. In response to EEP treatment, chondrocytes stimulated with IL-1β significantly decrease the protein expression of LC3I, ATG5, and AKT1 (Figure 4a–c). This result was also observed in mRNA expression of LC3I and ATG5 (Figure 4d,e). This suggests that EEP treatment, which is a rich mixture of polyphenols, particularly pinocembrin, could be able to return to it basal levels of autophagy. Recently, it has been reported that Chinese propolis reduce the inflammation through an inhibition of autophagy by reducing the distribution and accumulation of LC3 in vascular endothelial cells [83]. Another study mentions that a Taiwanese green propolis partially inhibited the NLRP3 inflammasome via autophagy induction [84]. Moreover, chrysin, another polyphenol, has been reported to be present in propolis and our samples (Table 1, number 32). Chrysin would be able to decrease the induction of proteins associated with autophagy in mesangial cells that were exposed to advanced glycation end products [85].

This decrease could restore this pathway normal levels, returning the homeostasis that had been lost with the inflammatory stimulus. As oxidative stress could mediate an excessive induction of autophagy [86], we analyzed whether EEP treatment, by exerting its scavenger action [62,63] could decrease the amount of NO that was released to the medium. Indeed, the EEP treatment reduced the amount of NO produced (Figure 6). Therefore, this could be the mechanism by which the autophagy activation may be inhibited, decreasing the expression of proteins, such as LC3 I, ATG5, and AKT1 (Figure 4a–c). To elucidate this, functional analysis should be done in the pathway of autophagy in chondrocytes in response to EEP treatment.

Collagen(s) are long-lived proteins [87,88]. Oxidative damage is, by far, the most important way of inducing a post-translational chemical modification of ECM of the cartilage that will alter collagen longevity [88]. So, a reduction in the oxidative damage by EEP treatment could improve the expression of healthy cartilage marker. EEP treatment induces an increase in protein expression of Col2a1 in chondrocytes that were stimulated with IL-1β probably through this way (Figure 5a,b). It has been described that the increase of ROS contributes to the degradation of the cartilage by means of an up regulation of MMPs [18] and that pinocembrin (one of the most abundant components of our propolis) inhibits the expression of MMP-1, MMP-3, and MMP-13 at the protein and mRNA levels in cartilage [66]. EEP treatment significantly reduces MMP-13 protein expression in chondrocytes that were stimulated with IL-1β (Figure 5c,d), probably again through an antioxidant mechanism. These results suggest the beneficial effects of EEP treatment in chondrocytes with OA in vitro, increasing the expression of healthy cartilage marker and reducing the OA marker.

To the best of our knowledge, there is no study that analyzes the effect of propolis on the expression of col2a1 or MMP13 on chondrocytes. Although, it has been described that pinocembrin was able to reduce MMP13 levels in chondrocytes that were stimulated with TNF-α [66] and chrysin was able to increase the levels of collagen II and reduce the levels of MMP13 in chondrocytes [85].

Koussounadis et al. [89], in 2015, mentioned that mRNA and protein expression do not always present good correlation for diverse reasons, for example, the different post-transcriptional regulation, mRNA degradation, the half-life of the protein, among others. In the case of the differences that were observed in the expression of Col2a1, we can infer that, since it is a long-lived protein, the oxidative stress that was generated by the inflammatory stimulus could favor the degradation of this protein. Moreover, its degradation would reduce by decreasing the amount of nitric oxide without altering mRNA expression when the treatment with EEP is applied (Figure 5a). It is also necessary to mention that the MMP13 expression has a negative relationship with the Col2a1 expression and since this metalloprotinase specifically degrades Col2a1, this could be another way in which the protein expression of Col2a1 varies without generating any modification of its mRNA.

Autophagy is a dynamic pathway whose activity can change in response to different stimulus, such as drugs, cell type, and confluence [7], because of that, it would be not unusual to find differences between gene and protein expression and sometimes it can be difficult to interpret the results. Hence, this is why we only mentioned the increase or reduction of protein expression.

According to the guidelines for the study of autophagy, most of the ATG genes do not show significant changes in mRNA levels when autophagy is induced [7]. A slight increase of mRNA of LC3 has been observed between 4 to 16 h after starvation, which decreases over time [90]. For this reason, it is thought that the increases in the mRNA expression of LC3 can be quite modest and are cell type and organism dependent. It is also suggested that the study of protein expression would be a more significant parameter in relation to the initiation and completion of autophagy [7]. Therefore, it is likely that, during the time in which the mRNA analysis was performed (24 h post treatment), it had already been degraded by intracellular mechanisms, which would be observed as a lower mRNA expression, despite the fact that the protein expression would remain high (Figure 4). Alternatively, we should have measured the rate of general protein breakdown by autophagy, to measure the autophagic flow, and therefore estimate the activity of the autophagy pathway. This is another limitation of this study.

It is important to consider that the antioxidant capacity of propolis responds to a heterogeneous mixture of compounds, including several polyphenols [72,73]. Each one possesses a particular antioxidant capacity and, for this reason, only the amount present of a particular polyphenol in the EEP is not a good indicator of the scavenger capacity of the compound [91]. For example, quercetin found in the chromatographic profile of Figure 1, as indicated with the number 11, has approximately 20 times more antioxidant power than pinocembrin, although it is found in a smaller quantities than this latter [91]. Therefore, we cannot rule out that the other polyphenols may be responsible for the antioxidant effect of propolis. In addition, we must take into account that the mixture of polyphenols could have an additive effect. In order to clarify this, the experiments should be carried out to include the effect of each of the different polyphenols that were present in the EEP sample, in comparison to the complete mixture.

**Figure 7:**
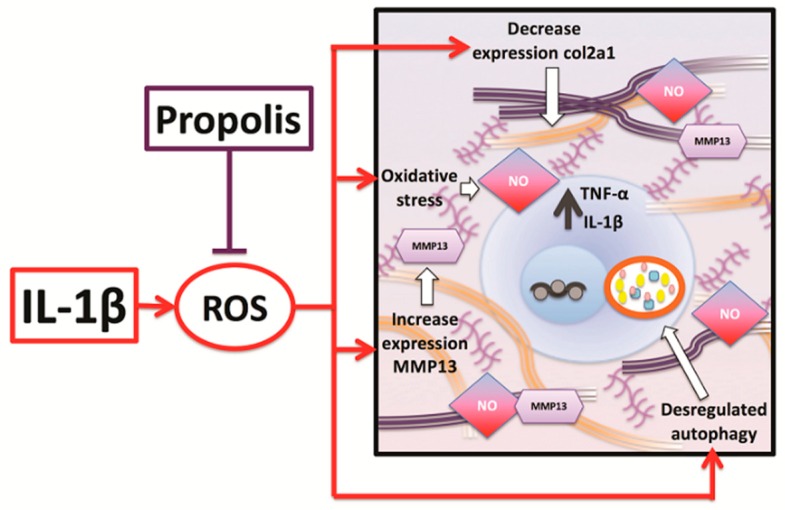
Mechanism proposed by which EEP treatment can reverse the alterations generated by the inflammatory stimulus in chondrocytes. Defects that are generated by the inflammatory stimulation in chondrocytes such as decrease expression of col2a1, increase of nitric oxide production, increase of MMP13 expression and deregulation of autophagy proteins are inhibited by EEP treatment through an antioxidant mechanism.

In conclusion, the inflammatory stimulation with IL-1β applied on chondrocytes causes a decrease in the expression of healthy cartilage marker and increases in the OA marker, also generates a deregulation of the autophagy pathway. The treatment with EEP is probably able to inhibit these deregulations counteracting the decompensation of the mechanisms that maintain cellular homeostasis, such as autophagy, which could be the initial trigger of mechanical and structural alterations of the tissue. We propose that the mechanism that is involved in the effect of propolis is through a reduction of oxidative stress that is generated by the application of the inflammatory stimulus (Figure 7).

Finally, we propose that treatment with dietary polyphenols in people with OA triggered by aging could be an effective complementary therapeutic approach, since, through anti-inflammatory, antioxidant, and autophagy regulating mechanisms, could inhibit or reduce the causes that would origin cartilage degeneration in relationship to age, such as: modification of the composition of the matrix, increase in oxidative stress, decrease in the number of chondrocytes that are associated with age, and change of the chondrocyte phenotype, among others, thus promoting the viability of chondrocytes and the maintenance of a healthy cartilage. More in vitro and in vivo studies are needed in order to support the effect of polyphenols in OA, evaluating the bioavailability and establishing an effective dose.

## 4. Materials and Methods

### 4.1. Preparation and Characterization of Ethanolic Extract of Propolis

A crude brown propolis sample was obtained from a mountainous area (latitude −38°58′4046′′, longitude −72°1′1573′′) near Cunco city, La Araucanía, Chile. Briefly, crude propolis was mixed with ethanol 80% in a 1:3 *w*/*v* proportion in an amber colored bottle and incubated for 30 min at 60 °C under constant mixing. Subsequently, the mixture was filtrated on a Whatman No. 1 filter paper in order to separate the ethanolic extract from crude propolis residues. The extract was left at 4 °C and then centrifuged for one night, in order to promote the precipitation of waxes and other poorly soluble waste. Subsequently, the propolis solvents were removed by evaporation and then the product was lyophilized and reconstituted in a 1:1 *w*/*v* proportion with ethanol. The EEP was analyzed with HPLC equipment (LC-20AD pumps, SIL-20AC injector, CTO-20A columns and DAD detector SPD-M20A, Kyoto, Japan); MS: MicroTOF-QII, Bruker Daltonics (Billerica, MA, USA). In the mass spectrometer, the DAD detector effluent was divided into a 1:10 ratio (split 1:10), with one part (50 μL/min.) being directed to the mass spectrometer. The electrospray source was used in negative mode at 3000 V. Nebulizer gas (nitrogen): 35 psi; drying gas (nitrogen): 6 L/min. at 220 °C. The mass scale of the equipment was calibrated with a sodium acetate solution. Additionally, the amount of pinocembrin was quantified while using the same method.

### 4.2. Cartilage Samples Collection and Primary Chondrocyte Culture

Normal articular cartilage was collected from five white New Zealand buck rabbits, which were anesthetized with overdose of propofol and euthanized using Potassium chlorate 60 mg/Kg. After knee joint surgery, the pieces of articular cartilage were dissected and separated from the underlying bone and connective tissues. The pieces were washed three times with PBS 1× and 5% penicillin/streptomicin. The extracted cartilage was digested in a solution of 2 mg/mL Protease Type XIV. Bacterial from (*Streptomyces griseus*) (Sigma Aldrich, St Louis, MO, USA) in PBS 1× for 1.5 h and 1.5 mg/mL collagenase B (Roche, Meylan, France) in basic medium DMEM at 37 °C overnight. This suspension was centrifuged at 1200 rpm for 8 min. to collect the chondrocytes and was washed with basic medium DMEM. The chondrocytes were cultured in DMEM/F12 (1:1 with 15% FBS plus 1% antibiotic mixture of penicillin/streptomycin) at a density of 1 × 10^5^ cells/mL and incubated in a humidified atmosphere of 5% CO_2_ at 37 °C. Culture medium was changed every two days and each passage was made when the confluence reached between 80–90%. We only used the second passage of cells in all experiments in order to avoid loss of chondrocyte phenotype [92].

### 4.3. Cell Viability Analysis in Response to EEP Treatment

Cell viability was assessed while using trypan blue staining, as previously described [93]. Rabbit chondrocytes were briefly incubated and then exposed to different concentrations of EEP (0, 2.5, 5, 7.5, 10, and 15 µg/mL) in 24 well plates at a density of 2 × 10^4^ cell per well for 24 h. Each experiment was performed in triplicate. The results are expressed as % of viable cells relative to control cells (untreated).

### 4.4. In Vitro Model of OA and Treatments

For the induction of OA-like biological changes, the chondrocytes were stimulated using IL-1β [17]. The cells were incubated for 24 h under the following conditions: Control (untreated); RAP (100 nM rapamycin, Sigma Aldrich, USA); BAF (20 nM bafilomycin, Sigma Aldrich, USA); IL-1β (10 ng/mL IL-1β); EEP (EEP 2.5 μg/mL); IL-1β and EEP (10 ng/mL IL-1β and 2.5 μg/mL EEP); and, vehicle (2% ethanol).

### 4.5. Western Blot Analysis

The chondrocytes were isolated from confluent monolayer cultures using RIPA buffer supplemented with 1mg/mL Halt™ Protease and Phosphatase Inhibitor Cocktail (Thermo Fisher, Waltham, MA, USA) at 4 °C for 30 min. The samples were then centrifuged at 15,000 rpm for 30 min., and the supernatants were harvested to measure the protein concentration. Proteins were quantified while using the Bradford method with BCA detection kit and adjusted to equal concentrations (45 µg) across different samples. Equal amounts of protein were heated at 95 °C for 5 min. and separated using 4–20% Mini protean TGX Precast gel (Biorad, Hercules, CA, USA). Following electrophoresis, the proteins were transferred onto a polyvinylidene fluoride membrane (PVDF, MilliporeSigma, Burlington, MA, USA). The membranes were blocked with 5% BSA (TBST) at room temperature for 1h and then incubated overnight at 4 °C with primary antibodies of autophagy proteins: ATG5 (ab108327, Abcam, Cambridge, UK), AKT1 (#9272, Cell Signaling, Danvers, MA, USA), LC3 (ab128025, Abcam, Cambridge, UK), and cartilage markers COL2A1 (ab34712, Abcam, Cambridge, UK), MMP13 (ab84594, Abcam, Cambridge, UK). Following three wash steps with TBST the membranes were incubated with Horseradish peroxidase (HRP) goat anti-rabbit IgG (#7074 Cell Signaling, Danvers, MA, USA) for 1 h at room temperature. They were washed then with TBST, three times, for 5 min. each time. Protein bands were detected using Amersham ECL TM Advance Western Blotting Detection Kit (GE Healthcare, Chicago, IL, USA). 

In the case that proteins have a weight similar to β-actin, stripping was performed with Restore Western Blot Stripping Buffer (21059, Thermo Fisher Scientific, Waltham (MA), USA) according to the manufacturer’s instructions. Finally, β-actin expression was used as load control in all western blot assays (A5441, Sigma Aldrich, St Louis, MO, USA). The quantification was performed by densitometry and bands analysis with the ImageJ 1.8.0 software (Rasband, W.S., ImageJ, U. S. National Institutes of Health, Bethesda, MD, USA)

### 4.6. Reverse Transcription-Quantitative Polymerase Chain Reaction (RT-qPCR)

Total RNA was isolated while using mirVana™ miRNA Isolation Kit (AM1560, Thermo Fisher Scientific, Waltham (MA), USA), according to the manufacturer’s instructions. First-strand cDNA was synthesized while using kit Superscript VILO cDNA synthesis, according to the manufacturer’s instructions, and Quantitative PCR was performed using 7500 real time PCR system with SYBR Green Master Mix (4309155, Thermo Fisher Scientific, Waltham (MA), USA). The forward and reverse primers that were used are shown in Table 2. β-actin expression was used as an internal control. Each experiment was repeated three times in technical triplicate. The relative expression of target genes was calculated while using the −2^−∆∆Cq^ method.

### 4.7. Measurement of NO Release

The NO release was measured from supernatant of rabbit chondrocytes cultures employing a NO chemiluminescence analyzer (model NOA, Sievers Instruments, Boulder, CO, USA). The evaluated conditions were: control; RAP; BAF; IL-1β; EEP; IL-1β and EEP; and, vehicle. All conditions were cultured for 24 h. The release of gaseous compounds was monitored for at least 8 h at intervals of 15 and 30 min., and 1, 2, 4, 6, and 8 h. After 15 min. of incubation, aliquots (0.5 mL) of accumulated gaseous materials in the headspace were injected into the detector chamber using a Hamilton Gastight syringe [94]. Each experiment was repeated three times.

### 4.8. Statistical Analysis

All of the experiments were repeated at least three times. The results were expressed as mean ± S.D., and the data was analyzed using one-way ANOVA followed by Dunnett, Bonferroni or Tukey Multiple Comparisons while using Sigma Plot (Analysis made in Graph Pad Prism version 5) to determine any significant differences. *p* < 0.05 was considered to be statistically significant.

## Figures and Tables

**Table 1:** Identification data of compounds detected in the Ethanolic Extract of Propolis.

Nº	R_t_ (Min)	λ_max_ (nm)	[M-H]^−^ (*m*/*z*)	Ion Formula	Error (ppm)	Compound
**1**	7.3	292, 323	179.0353	C_9_H_7_O_4_	1.6	Caffeic acid
**2**	11.4	310	163.0399	C_9_H_7_O_3_	−2.2	*p*-Coumaric acid
**3**	12.6	298, 320	193.0508	C_10_H_9_O_4_	0.9	Isoferulic acid
**4**	14.3	295, 322	193.0506	C_10_H_9_O_4_	0.0	Ferulic acid
**5**	16.8	292	287.0565	C_15_H_11_O_6_	1.3	Dihydrokaempferol
**6**	18.0	230	121.0281	C_7_H_5_O_2_	−6.1	Benzoic acid
**7**	22.4	295, 320	207.0658	C_11_H_11_O_4_	0.3	3,4-Dimethyl-caffeic acid
**8**	30.4	295, 322	207.0664	C_11_H_11_O_4_	0.0	3,4-Dimethyl-caffeic acid isomer
**9**	31.2	285	285.0768	C_16_H_13_O_5_	−0.2	Pinobanksin-5-methyl ether
**10**	32.3	308	177.0559	C_10_H_9_O_3_	1.0	*p*-Coumaric acid methyl ester
**11**	33.3	255, 369	301.0351	C_15_H_9_O_7_	−0.8	Quercetin
**12**	34.3	291	271.0619	C_15_H_11_O_5_	2.5	Pinobanksin
**13**	37.6	252, 350	285.0404	C_15_H_9_O_6_	−0.1	Luteolin
**14**	39.5	256, 357	315.0504	C_16_H_11_O_7_	−1.9	Quercetin-3-methyl ether
**15**	44.0	265, 366	285.0410	C_15_H_9_O_6_	1.8	Kaempferol
**16**	44.6	286	269.0826	C_16_H_13_O_4_	2.7	Pinocembrin-5-methyl ether
**17**	46.3	267, 338	269.0448	C_15_H_9_O_5_	2.6	Apigenin
**18**	46.9	254, 370	315.0510	C_16_H_11_O_7_	−0.1	Isorhamnetin
**19**	49.7	266, 352	299.0549	C_16_H_11_O_6_	−4.0	Kaempferol-methyl ether
**20**	50.5	311	173.0621	C_11_H_9_O_2_	7.6	Chrysin-5-methyl ether
**21**	50.8	265, 310	267.0663	C_16_H_11_O_4_	−3.2	Cinnamylidenacetic acid
**22**	51.2	290	329.0674	C_17_H_13_O_7_	2.1	Unknown
**23**	53.6	296	313.0714	C_17_H_13_O_6_	1.1	Pinobanksin acetate derivative
**24**	55.3	255, 369	315.0508	C_16_H_11_O_7_	−0.7	Rhamnetin
**25**	57.1	290	255.0664	C_15_H_11_O_4_	0.5	Pinocembrin
**26**	59.4	327	269.0818	C_16_H_13_O_4_	0.4	Caffeic acid benzyl ester
**27**	59.8	300, 325	247.0977	C_14_H_15_O_4_	0.7	Caffeic acid isoprenyl ester (isomer)
**28**	60.3	290	285.0766	C_16_H_13_O_5_	−0.8	Unknown
**29**	61.1	292	313.0727	C_17_H_13_O_6_	3.1	Pinobanksin-3-*O*-acetate
**30**	61.4	300, 325	247.0988	C_14_H_15_O_4_	4.9	Caffeic acid isoprenyl ester (isomer)
**31**	62.0	301, 325	247.0986	C_14_H_15_O_4_	3.9	Caffeic acid isoprenyl ester (isomer)
**32**	63.3	268, 314	253.0515	C_15_H_9_O_4_	3.6	Chrysin
**33**	65.7	298, 325	283.0987	C_17_H_15_O_4_	4.0	Caffeic acid phenylethyl ester (CAPE)
**34**	66.5	265, 359	269.0467	C_15_H_9_O_5_	4.5	Galangin
**35**	69.3	268, 332	283.0624	C_16_H_11_O_5_	4.2	Acacetin
**36**	69.8	311	253.0883	C_16_H_13_O_3_	5.0	p-Coumaric acid benzyl ester
**37**	70.4	298, 325	283.0987	C_17_H_15_O_4_	3.9	Caffeic acid derivative (phenylethyl ester)
**38**	70.9	298, 323	261.1138	C_15_H_17_O_4_	2.3	Caffeic acid derivative (hexenoate ester)
**39**	72.2	298, 324	261.1145	C_15_H_17_O_4_	4.9	Caffeic acid derivative (hexenoate ester)
**40**	72.7	290	327.0904	C_18_H_15_O_6_	9.0	Pinobanksin-3-*O*-propionate
**41**	74.1	247, 295, 325	295.0993	C_18_H_15_O_4_	5.7	Caffeic acid cinnamyl ester
**42**	75.7	298	267.1033	C_17_H_15_O_3_	2.3	Unknown
**43**	76.2	300, 327	269.0825	C_16_H_13_O4	2.0	Caffeic acid derivative
**44**	78.7	289	433.1313	C_25_H_21_O_7_	4.7	Pinocembrin methoxyphenylpropionate derivative
**45**	82.4	nd	417.1376	C_25_H_21_O_6_	7.9	Pinobanksin phenylpropionate derivative
**46**	82.9	286	353.1062	C_20_H_17_O_6_	9.0	Pinobanksin-3-*O*-pentenoate
**47**	83.3	293	341.1048	C_19_H_17_O_6_	8.2	Pinobanksin-3-*O*-butyrate/isobutyrate
**48**	88.3	nd	311.2249	C_18_H_31_O_4_	6.8	Unknown
**49**	92.5	292	355.1220	C_20_H_19_O_6_	9.3	Pinobanksin-3-*O*-pentanoate/2-methylbutyrate
**50**	96.1	291	403.1238	C_24_H_19_O_6_	12.6	Pinobanksin-3-*O*-phenylpropionate
**51**	97.7	291	369.1397	C_21_H_21_O_6_	14.4	Pinobanksin-3-*O*-hexanoate
**52**	99.6	283	293.2148	C_18_H_29_O_3_	8.7	Unknown
**53**	103.6	283	281.2509	C_18_H_33_O_2_	9.5	Unknown

Rt: Retention time; λmax: Maximum wavelength; *m*/*z*: Mass-to-charge ratio.

**Table 2:** Primers used to analyze gene expression in rabbit chondrocytes.

Gene Symbol	Sequence Forward (5′-3′)	Sequence Reverse (5′-3′)
COL2A1	GGT GAC TAC TGG ATA GAC CCC AAC CAA	TGA AGT GGA AGC CGC CAT TGA TG
MMP13	GAA TTA AGG AGC ATG GCG AC	TAA GGA GTG GCC GAA CTC AT
ATG5	CGT CCT GTG GCT GCA GAT G	AAG GAC ACA CTT CTT TGA GGA GAT C
MAPLC3A/B (LC3I/II)	GCC TTC TTC CTG CTG GTG AAC	AGC CGT CCT CGT CTT TCT CC
AKT 1	ATG GCA CCT TCA TTG GCT AC	CCC AGC AGC TTC AGG TAC TC
ACTB	AGA CCA CCT TCA ACT CGA TCA T	ACT CGT CAT ACT CCT GCT TGC T

## Abbreviations

AKT	Serine/Threonine Kinase
ATG	Autophagy-related proteins
ATG5	Autophagy related 5
BAF	Bafilomycin
BECN1	Beclin1
Col2a1	Collagen type II alpha 1 chain
ECM	Extracellular matrix
EEP	Ethanolic extract of propolis
HPLC	High-performance liquid chromatography
HRP	Horseradish peroxidase
iNOS	Inducible nitric oxide synthase
IL-1β	interleukin 1 beta
LC3	Microtubule associated protein 1 light chain 3 alpha (MAP1LC3A)
mRNA	Messenger RNA
MMPs	Matrix metalloproteinases
MMP-13	Matrix metalloproteinase 13
mTOR	Mammalian target of rapamycin
mTORC-1	Mammalian target of rapamycin complex 1
NO	Nitric oxide
OA	Osteoarthritis
OARSI	Osteoarthritis Research Society International
PB	Pinocembrin
RAP	Rapamycin
RNOS	Reactive Nitrogen and Oxygen Species
TNF-α	Tumor necrosis factor α
ULK1	unc-51 like autophagy activating kinase 1
